# Catatonia Associated With First-Time Psilocybin Exposure: A Case Report

**DOI:** 10.7759/cureus.106474

**Published:** 2026-04-05

**Authors:** Jasbir Singh, Pranathi Mruthyunjaya

**Affiliations:** 1 Psychiatry, Contra Costa Regional Medical Center, Martinez, USA

**Keywords:** bush-francis catatonia rating scale, catatonia, dsm-5 diagnostic criteria, magic mushroom, psilocybin, psychosis

## Abstract

In this case report, we highlight a 28-year-old male patient with predominantly retarded type of catatonia following a single incidence of psilocybin ingestion. This patient presented with a history of six months of mutism and inability to feed himself after consuming psilocybin for the first time. He did not have any history of psychiatric illness or substance use. Imaging and laboratory results were unremarkable. The goal of this case report is to document one of the earliest records of psilocybin-induced catatonia.

## Introduction

Catatonia of the retarded type is a behavioral syndrome characterized by rigidity, immobility, mutism, staring, and other clinical signs. Catatonia is observed in many psychiatric and non-psychiatric illnesses. One study revealed that approximately 10% of acutely ill patients in an inpatient psychiatric unit had catatonia with associated mood disorders [1].

Catatonia is defined in the Diagnostic and Statistical Manual of Mental Disorders, 5th Edition (DSM-5), as having "three or more of 12 psychomotor features in the diagnostic criteria for catatonia associated with another mental disorder and catatonic disorder due to another medical condition" [2]. Commonly observed symptoms include waxy flexibility, catalepsy, mutism, stupor, negativism, posturing, stereotypy, echolalia, grimacing, echopraxia, and agitation that are not influenced by external stimuli. The Bush-Francis Catatonia Rating Scale is widely used to measure the severity of catatonia. Each item is scored on a scale of 0-3 depending on severity, and higher scores indicate worse severity [3].

There are three subtypes of catatonia: retarded, excited, and malignant. The common symptoms observed in the retarded type are immobility, intense staring, posturing, negativism, and mutism [4]. For centuries, schizophrenia patients have been divided into two different groups: the catatonia group and the non-catatonia group [5]. In the DSM-5, catatonia is described as a separate illness mostly associated with mood disorders such as bipolar I and II disorders, schizophrenia spectrum disorders, and autism spectrum disorders [1]. Complications such as malnutrition, dehydration, contractures, pressure ulcers, deep venous thromboses, and pulmonary embolisms are commonly seen in patients with catatonia [6].

The lorazepam challenge is a common diagnostic tool for catatonia. A patient is given 1-2 mg of lorazepam intramuscularly, intravenously, or orally and observed for rapid resolution of symptoms after a 20- to 30-minute time [4]. Research aimed at understanding the difference between illicit substances and catatonic episodes is minimal, with one of the only studies conducted analyzing specifically the temporal relationship between substance use and catatonia [7]. Our case can lead to proper awareness of substance-induced catatonia. Consent to publish the case history was obtained from the patient.

While catatonia has been extensively described in the context of primary psychiatric disorders, literature examining its association with psilocybin use is comparatively limited. As psilocybin gains increasing attention in research and therapeutic applications, understanding potential adverse neuropsychiatric outcomes is essential. 

## Case presentation

A 28-year-old male patient with no prior psychiatric history, substance use, medical comorbidities, or family history of psychotic disorders presented with a six-month course of profound mutism and severe self-neglect, including refusal to eat or engage in personal hygiene. The patient's symptoms started after he consumed 3.4 g of psilocybin six months before hospitalization. He was introduced to psilocybin by a friend, and he decided to try it out of curiosity. Upon consumption, the patient was silent and reflective for a few hours before going missing. The patient was found alone in a nearby park about 24 hours later, in a disorganized state, and was taken home by family. After returning home, the patient was nonverbal, routinely sleeping 16 hours a day, and regularly aggressive with his family. The patient was unable to feed or groom himself and was fed by family members. He did not receive any medical care, and he was taken care of by his family during this time. These symptoms persisted for six months when the family decided to call for help.

Emergency services were requested due to the patient's continued catatonic presentation and aggressive episodes. The patient was aggressive when interacting with paramedics and was administered 15 mg of midazolam intramuscularly en route to the hospital. In the inpatient unit, the patient presented with a stuporous, almost mute, and intermittently agitated catatonic state. He was uncooperative during the safety check and exhibited physical and verbal agitation, which was treated with 10 mg of ziprasidone and 2 mg of lorazepam intramuscularly. All the medications were administered intramuscularly because his continuous psychosis and physical agitation made him unable to accept oral medication. An initial diagnosis of catatonia was established, with a score of 19 on the Bush-Francis Catatonia Rating Scale. Itemized scores were as follows: immobility/stupor (2), mutism (3), staring (2), grimacing (1), stereotypy (1), negativism (3), withdrawal (3), impulsivity (2), and combativeness (2). A lorazepam challenge was performed. After 30 minutes, the patient responded to 1 mg of lorazepam by mouth and was significantly more conversant, ambulatory, and more likely to respond appropriately to the assessment questions. His subsequent Bush-Francis Catatonia Rating Scale score decreased to 10, with significant improvement in immobility, mutism, negativism, impulsivity, and combativeness. Itemized scores were as follows: immobility/stupor (1), mutism (0), staring (2), grimacing (1), stereotypy (1), negativism (2), withdrawal (2), impulsivity (1), and combativeness (0).

In the psychiatric unit, the patient was adherent to psychotropic medications and was given 2 mg of lorazepam daily and 2 mg of risperidone daily, with an observed resolution of catatonic symptoms. After nine days of medical treatment, the patient had regained speech and emotional expression and was able to feed and groom himself. His MRI and laboratory results were unremarkable. He was connected to outpatient psychiatry services for follow-up care. However, he was subsequently lost to follow-up. 

## Discussion

While there is moderate documentation of hallucinogen-induced psychosis [8], such as N,N-dimethyltryptamine (DMT)-induced psychosis [9] and lysergic acid diethylamide (LSD)-induced catatonia [10], there are a limited number of documented cases of catatonia/psychosis induced by psilocybin. Recognizing catatonic characteristics and diagnosing patients accurately will allow clinicians to proceed with treatment. But there is a need to understand potential differences in recovery outcomes between substance-induced and non-substance-induced catatonia.

The relationship between psilocybin and catatonia is currently understudied. Psilocybin mainly acts via agonism of cortical 5-hydroxytryptamine receptor 2A (5-HT2A) receptors. These serotonergic effects have downstream consequences on dopaminergic and GABAergic systems. Stimulation of 5-HT2A receptors has been shown to modulate mesolimbic and mesocortical dopamine release while influencing gamma-aminobutyric acid (GABA) interneurons that regulate cortical excitability. Catatonia has been hypothesized to arise from dysregulation within GABAergic, dopaminergic, and glutamatergic circuits, with evidence pointing towards impaired GABAergic inhibition and altered dopaminergic signaling in frontal-subcortical pathways. This is supported by the robust clinical response to benzodiazepines, which enhance GABA_A receptor activity [11,12].

In this case report, the patient showed significant improvement in his catatonic symptoms after the administration of lorazepam. It is unclear whether the time elapsed in a catatonic state before receiving treatment may have affected the patient's recovery. This case report highlights the importance of considering substance use when treating a catatonic patient. Moreover, a systematic review and meta-analysis of clinical trials support that psilocybin may be an effective treatment option for treatment-resistant depression [10]. One study revealed that a larger, single dose of psilocybin significantly reduced depression scores more than did smaller doses of psilocybin over three weeks. However, the associated adverse effects of a larger dose of psilocybin necessitate further research as a treatment option [13]. This patient's psilocybin-induced catatonia suggested that patients treated with psilocybin for depression should be closely monitored. Another study described a patient with catatonia secondary to schizoaffective disorder, depressive type, developing bilateral deep venous thrombosis progressing to a saddle pulmonary embolism without any predisposing factors to hypercoagulability other than immobility and obesity [14]. 

Future research could also investigate characteristic differences between substance-related and non-substance-related catatonic episodes and their treatment responses.

## Conclusions

This case report is the first documentation of psilocybin-induced catatonia following an ingestion of a single dose of psilocybin and can serve as a reference point for future cases. This patient presented with predominantly standard characteristics of retarded-subtype catatonia and no prior mental health history. He was adequately treated for catatonia using benzodiazepine. However, there is a need to understand how comorbid conditions may affect treatment options for psilocybin-induced catatonia. 

## References

[1] Taylor MA, Fink M (2003). Catatonia in psychiatric classification: a home of its own. Am J Psychiatry.

[2] (2022). Diagnostic and Statistical Manual of Mental Disorders, Fifth Edition, Text Revision (DSM-5-TR).

[3] Bush G, Fink M, Petrides G, Dowling F, Francis A (1996). Catatonia. I. Rating scale and standardized examination. Acta Psychiatr Scand.

[4] Daniels J (2009). Catatonia: clinical aspects and neurobiological correlates. J Neuropsychiatry Clin Neurosci.

[5] Fink M (2011). Catatonia from its creation to DSM-V: considerations for ICD. Indian J Psychiatry.

[6] Yeoh SY, Roberts E, Scott F, Nicholson TR, David AS, Rogers JP (2022). Catatonic episodes related to substance use: a cross-sectional study using electronic healthcare records. J Dual Diagn.

[7] Hermle L, Kovar KA, Hewer W, Ruchsow M (2008). Hallucinogen-induced psychological disorders [Article in German]. Fortschr Neurol Psychiatr.

[8] Brown T, Shao W, Ayub S, Chong D, Cornelius C (2017). A physician's attempt to self-medicate bipolar depression with N,N-dimethyltryptamine (DMT). J Psychoactive Drugs.

[9] Prajapati Ramprasad P, Fabiu D, Pradhan B (2016). Clinical course and management of drug-induced catatonia and paranoid behavior in an adolescent. Psychiatry Clin Psychopharmacol.

[10] Bowers MB Jr, Swigar ME (1983). Vulnerability to psychosis associated with hallucinogen use. Psychiatry Res.

[11] Walther S, Strik W (2016). Catatonia. CNS Spectr.

[12] Nichols DE (2016). Psychedelics. Pharmacol Rev.

[13] Gukasyan N, Davis AK, Barrett FS, Cosimano MP, Sepeda ND, Johnson MW, Griffiths RR (2022). Efficacy and safety of psilocybin-assisted treatment for major depressive disorder: prospective 12-month follow-up. J Psychopharmacol.

[14] Maner BS, Singh J, Camacho H, Jenson AK (2021). Catatonia-induced saddle pulmonary embolism. Cureus.

