# Correction: Catatonia Associated With First-Time Psilocybin Exposure: A Case Report

**DOI:** 10.7759/cureus.c439

**Published:** 2026-05-27

**Authors:** Jasbir Singh, Pranathi Mruthyunjaya

**Affiliations:** 1 Psychiatry, Contra Costa Regional Medical Center, Martinez, USA

This article has been corrected as follows: the third sentence of the case presentation, “The patient's symptoms started after he consumed 3.4 mg of psilocybin six months before hospitalization", has been revised to the correct dose of 3.4 g.

